# Resin-modified glass ionomer containing calcium glycerophosphate: physico-mechanical properties and enamel demineralization

**DOI:** 10.1590/1678-7757-2018-0188

**Published:** 2019-02-21

**Authors:** Sâmia Sass SANTOS, Alberto Carlos Botazzo DELBEM, João Carlos Silos MORAES, José Antônio Santos SOUZA, Lenara Queiroz Chaves OLIVEIRA, Denise PEDRINI

**Affiliations:** 1 Universidade Estadual Paulista (UNESP), Faculdade de Odontologia, Departamento de Odontopediatria e Odontologia Social, Araçatuba, São Paulo, Brasil.; 2 Universidade Estadual Paulista (UNESP), Faculdade de Engenharia, Departamento de Física e Química, Ilha Solteira, São Paulo, Brasil.; 3 Universidade Estadual Paulista (UNESP), Faculdade de Odontologia, Departamento de Cirurgia e Clínica Integrada, Araçatuba, São Paulo, Brasil.

**Keywords:** Glass ionomer cements, Tooth demineralization, Polymerization, Compressive strength, Phosphates

## Abstract

Sources of calcium and phosphate have been added to dental restorative materials to improve their anticaries effect.

**Objective:**

This study evaluated the effect of adding calcium glycerophosphate (CaGP) to resin-modified glass ionomer cement (RMGIC) on the physico-mechanical properties, ion release, and enamel demineralization. Material and Methods: Specimens were fabricated for each experimental group: RMGIC without CaGP (Control), RMGIC with 1, 3 and 9% CaGP. To determine the release of fluoride (F), calcium (Ca) and phosphorus (P), six specimens were immersed in demineralization and remineralization solutions for 15 days. In another experimental trial, the following physico-mechanical properties were evaluated at time intervals of 1 and 7 days after fabrication: compressive strength (n=12), diametral tensile strength (n=12), surface hardness of material (n=6) and the degree of conversion of monomers (n=8). To study enamel demineralization, specimens (n=12) were attached to enamel blocks and submitted to pH-cycling. Subsequently, surface and cross-sectional hardness and the concentration of F, Ca and P in enamel were determined.

**Results:**

The addition of CaGP to RMGIC led to higher mean release of F, Ca and P when compared with control (p<0.001). Mechanical properties were within the range of those of the ionomer cements after addition of 1% and 3% CaGP. The degree of conversion did not differ between groups at the 1st and the 7th day (p>0.439). The addition of 3% and 9% CaGP reduced mineral loss and increased F, Ca and P in the enamel when compared with control (p<0.05).

**Conclusion:**

The addition of 3% CaGP in RMGIC increased the release of F, P and Ca, reduced enamel demineralization, and maintained the physico-mechanical properties within the parameters for this material.

## Introduction

Dental restorative materials with superior clinical performance regarding the occurrence of secondary carious lesions in primary teeth release fluoride ions (F^−^: nominated as F) into the oral medium.^1^ Among these, the glass ionomer cements (GICs) release large quantities of F and they are the material of choice for use in patients with high caries activity. Nevertheless, GICs have reduced fracture strength^2^ and esthetic appearance^3^ compared with composite resin. As the process of demineralization and remineralization depends on the presence of calcium (Ca^2+^: abbreviated as Ca) and phosphate (PO_4_^3−^: abbreviated as P) ions in the medium,^4^ compounds containing amorphous calcium phosphate stabilized by casein phosphopeptides (CPP-ACP) have been added to improve the anticariogenic potential of GIC material.^5^ These results were associated with the release of F, Ca and P ions by the GIC. Notwithstanding, the incorporation of CPP-ACP into the GICs decreased their diametral tensile and compressive strength.^5^

Another calcium phosphate with anticariogenic action, calcium glycerophosphate (CaGP), is an organic phosphate with affinity for tooth enamel. CaGP provides Ca and P ions increasing their levels into the plaque^6^ ^,^ ^7^ and with a plaque-pH buffering effect. In some studies, CaGP (50% α- and 50% β-isomer) has been added to low-fluoride toothpastes showing an improvement in their anticaries effect.^8^ ^-^ ^10^ This effect was related to its capacity of adsorption onto the enamel surface and increased ionic activity of neutral species, such as CaHPO_4_^0^ and HF^0^, in dental biofilm.^4^ ^,^ ^8^ The neutral species have a higher diffusion coefficient into enamel than that of charged species.^4^ Based on the above studies, the addition of CaGP to the GICs would be another alternative to increase their anticariogenic capacity.

Addition of new compounds to the GICs could lead to improvements in the material properties and provide greater understanding of the demineralization and remineralization processes in the presence of calcium phosphate and fluoride. From this aspect, it was considered important to verify whether the incorporation of CaGP in concentrations of 1%, 3% and 9% into a resin-modified glass ionomer cement (RMGIC) would influence the release of F, Ca and P, physico-mechanical properties (diametral tensile and compressive strength, surface hardness and degree of conversion) and demineralization of enamel (surface and cross-sectional hardness and F, Ca and P uptake). The null hypothesis of the study was that the addition of CaGP to the RMGIC would not alter the release of F, Ca and P, its physico-mechanical properties and the effect on enamel demineralization.

## Material and methods

### Preparation of the RMGIC mixture with CaGP

To standardize the particle size of CaGP microparticles (DL-50% α- and 50% β-isomer; CAS 1336-00-1, white powder, Sigma-Aldrich Co., St. Louis, MO, USA) at up to 53 µm in diameter, an electromagnet shaker and granulometric sieve for determining particle size were used (Bertel Indústria Metalúrgica LTDA, Caieiras, SP, Brazil). The RMGIC used in this study was Fuji II LC A2 (GC Corporation, Hasunuma-cho, Itabashi-ku, Tokyo, Japan) to which CaGP at concentrations of 1, 3 and 9%^10^ were added to RMGIC powder. For each 3.2 g of RMGIC powder: 0.0323 g, 0.0990 g and 0.3165 g of CaGP was added, respectively. The mixture (RMGIC powder and CaGP) was homogenized in an agate mortar (Planetary Micro Mill PULVERISETTE 7 classic line, Fritsch GmbH, Idar-Oberstein, Rhineland-Palatinate, Germany), in 5 cycles (normal/reverse) with duration of 1 minute each, at a speed of 100 rpm. The RMGICs were mixed by hand with powder-liquid ratio (3.2 g powder to 1.0 g of liquid), as recommended by manufacturer, for 2 minutes and 30 seconds.

### F, Ca and P release from RMGIC

#### Preparation of the specimens

Specimens (n=6) were made with RMGIC using a bipartite metal matrix (5 mm diameter and 2 mm thickness) defining 4 groups: RMGIC without CaGP (denominated as Control), RMGIC with 1% of CaGP (abbreviated as 1% CaGP), 3% of CaGP (abbreviated as 3% CaGP) and 9% of CaGP (abbreviated as 9% CaGP). A stainless-steel wire, 0.25 mm in diameter was positioned inside the matrix before inserting the materials to facilitate manipulation of the specimens. The upper and lower surfaces of each specimen were polymerized by light-emitting diode (LED) unit (Blue Star 2, Microdont, Socorro, SP, Brazil), with the light gun tip diameter of 7 mm, for 20 seconds.^11^ The light output intensity was periodically checked (1070 mW/cm^2^). After curing, the excess material was carefully removed.

#### pH-cycling for the release of F, Ca and P from RMGIC

The specimens, suspended by stainless steel wires, were randomly placed in test tubes with lids. Each tube contained 2 mL of demineralization (DE) or remineralization (RE) solutions. Initially, specimens were stored for 6 hours in DE solution (2.0 mmol/ L Ca and P, in acetate buffer 75 mmol/L, pH 4.7). Then specimens were placed in new test tubes containing the RE solution (1.5 mmol/L Ca, 0.9 mmol/L P, 150 mmol/L KCl in cacodylate buffer 20 mmol/L, pH 7.0) for 18 hours.^11^ Test tubes were subjected to constant shaking in an orbital shaker (TE-420 Orbital – Tecnal, Piracicaba, SP, Brazil) at 37°C. These procedures were repeated for 15 days. The specimens were dried with absorbent paper before being immersed in a new solution. Solutions were daily collected, identified and stored in test tubes at 4°C to measure F, Ca and P released from RMGICs.

#### Analysis of F, Ca and P in the DE and RE solutions

Fluoride concentration in DE and RE solutions was measured using a specific fluoride ion-selective electrode (Orion 9609-BN, Orion Research, Inc., Beverly, MA, USA) and a digital ion analyzer (Orion 720 A, Orion Research, Inc., Beverly, MA, USA), previously calibrated with standard solutions (0.125 to 16 mg F/mL). For the dosage of F, 0.5 mL of the DE and RE solutions were pipetted, and 0.5 mL of TISAB II were added. Readouts were performed under constant agitation in a magnetic agitator (TE-081, Tecnal, Piracicaba, SP, Brazil).

The Ca was measured using the Arsenazo colorimetric method.^12^ A 5 μL aliquot, in duplicate for both standard and sample solutions, to which 50 μL of deionized water and 50 μL of Arsenazo III were added. For calibration, standard solutions containing 40 to 200 mg Ca/mL were used. P was measured adding 40 μL of 50 mmol/L sulfuric acid and 40 μL of 1% periodic acid in 200 μL of DE and RE solution, maintained in a boiling bath for 1 hour.^13^ After cooling, 160 μL of deionized water was added and an aliquot of 55 μL was transferred to a 96-well plate. Subsequently, 10 μL of 8% sodium sulfite and 5 μL of 7% sodium molybdate were added. After homogenization of the reagents, 5 μL of 1% hydroquinone was added. The difference in phosphorus concentrations in the DE (70.4 ±3.2 µg P/mL) and RE (41.3 ±2.2 µg P/mL) solutions before and after acidic hydrolysis was considered as originating from the CaGP present in the solutions. Ca and P of the solutions were measured by spectrophotometric plate reader (Microplate Spectrophotometer EON, Biotek, Winooski, VT, USA) at a 650 nm and 640 nm wavelength, respectively.

The values were obtained in µg/mL and converted in µg/cm^2^, according to the area of the materials. F, Ca and P in the DE and RE solutions were determined separately and then the DE and RE solutions results were summed (DE+RE), completing a 1-day time interval and one pH-cycle, during the 15 days of the experiment.

## Measurements of physico-mechanical properties

### Diametral tensile and compressive strength

Twelve specimens of each experimental group were made for the diametral tensile (6 mm diameter × 3 mm height) and compressive strength (2 mm diameter × 4 mm height) tests.^14^ After time intervals of 1 and 7 days of storage at 37°C in a moist environment, the specimens were submitted to diametral tensile and compressive strength tests in an Instron universal test machine (DL3000, Instron Co., Canton, MA, USA), in horizontal and vertical positions, respectively, with a load cell of 500 N, at 1 mm/min crosshead speed, until fracture. The diametral tensile strength values (kgf/cm^2^) were calculated using the equation: 2F/πDT, where F is the failure load, D, the diameter; and T, the height of the specimens. Compressive strength values (kgf/cm^2^) were calculated by dividing the load (F) by cross-sectional area. All values were converted into MPa.

### Surface hardness analysis

**Figure 1:**
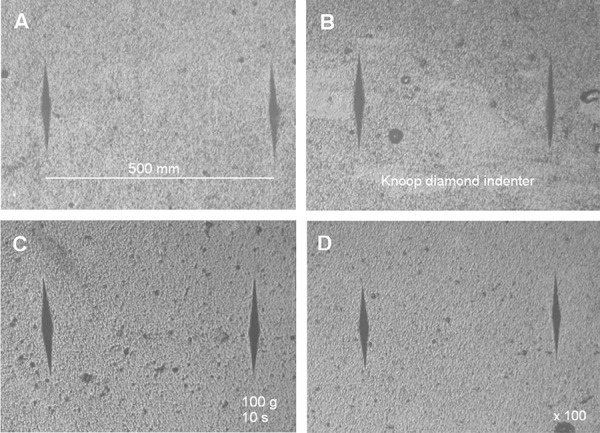
Photomicrographs of surface hardness test according to the groups at the 7th day: A: Control; B: 1% CaGP; C: 3% CaGP and D: 9% CaGP (x100 magnification)

Six disc-shaped specimens (5 mm diameter **×** 2 mm thickness)^11^ of each material were manufactured and maintained in relative humidity for 1 day. Subsequently, five indentations 500 µm equidistant from each other were made on the top surface (Figure 1), using a microhardness tester (Micromet 5114, Buehler, Lake Bluff, IL, USA), Knoop diamond indenter, 100 g load applied for 10 seconds.^11^ After this, the specimens were stored in relative humidity for 7 days, after which the hardness test was repeated.

### Degree of conversion (DC) of the monomers of RMGIC

**Figure 2:**
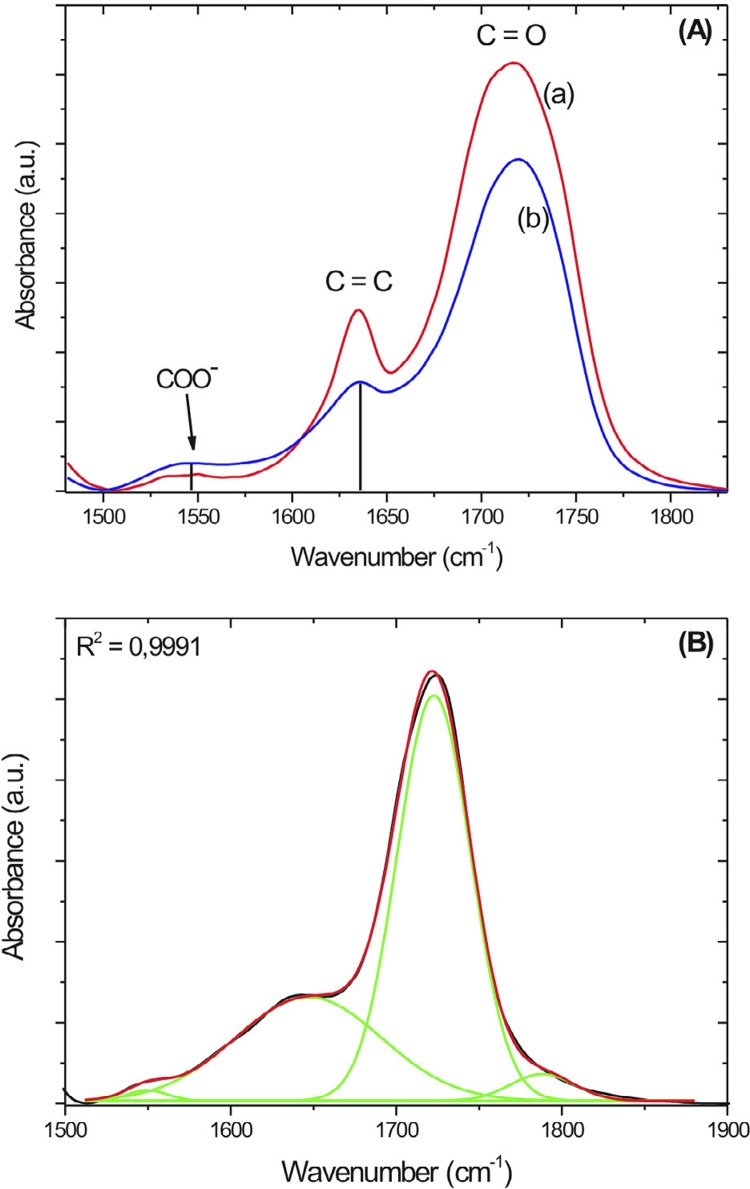
(A) RMGIC absorption band before (a) and after (b) polymerization; (B) Fit of the RMGIC+CaGP spectrum, buth in the region from 1500 to 1850 cm-1. The green lins in (B) represent four gaussian shapes and the red line their sum, which give the best fit to the experimental spectrum (black line)

RMGICs were manipulated (n=4/group), as previously described, inserted between two glass plates and pressed to form a film of approximately 0.12±0.02 mm. Specimens were polymerized by LED unit for 20 seconds on each side of the sample and were stored in a dry medium for 1 and 7 days at 37°C. After these time intervals, the RMGIC films were triturated and mixed with potassium bromide (KBr) in the ratio of 1:70 (mg:mg) to make tablets. The absorption spectra were obtained by the infrared transmission method (Fourier transform) in a spectrophotometer (Nexus 670, Nicolet Instrument Corporation, Madison, WI, USA) in the spectral region between 1400 and 2000 cm^-1^, using 128 scans and resolution of 4 cm^-1^. The liquid of the RMGICs was also mixed with KBr to obtain the absorption spectra of the monomer. DC was evaluated using the absorption band associated with aliphatic C=C bonds of the monomer, located at 1640 cm^-1^. The carboxylic acid COO^−^ absorption band, located at 1540 cm^-1^, was used as an internal standard of normalization (Figure 2). The absorption peak at 1720 cm^-1^ is attributed to C=O stretching vibration of the monomer. The percentage of unreacted aliphatic C=C bonds remaining throughout the polymerization reaction was obtained by the equation:

$$\mathrm{DC}(\%)=\left(1-\frac{\left[Abs\left(C=C\right)/Abs\left(COO^{-}\right)\right]polymer}{\left[Abs\left(C=C\right)/Abs\left(COO^{-}\right)\right]monomer}\right)\times 100.$$

In the RMGIC+CaGP spectra, the absorption peak at 1540 cm^-1^ is not well-defined. In these cases, it was necessary to fit the curves using Gaussian functions to evaluate the Abs(C=C)/Abs(COO^−^) ratio (Figure 2B).

## Demineralization and remineralization cycling

### Preparation of enamel blocks

Enamel blocks (4×4×3 mm) were prepared from freshly extracted bovine incisors and the enamel surface was ground flat, resulting in the removal of a depth of approximately 120 mm of the enamel.^15^ After polishing, cross-sections were cut at 1 mm from the border of the block to obtain 4×3×3 mm enamel slabs. For selection purposes, the initial surface hardness (SH_1_) was measured (Knoop) by making five indentations spaced 100 μm from each other, at a distance of 300 μm from the sectioned enamel border using a hardness tester (Micromet 5114, Buehler, Lake Bluff, IL, USA), with 25 g load applied for 10 seconds. The mean value was calculated and 60 enamel blocks with SH_1_ between 320 and 370 KHN were selected for the study.^15^

### pH-cycling test

Five experimental groups (n=12), mean surface hardness within the confidence interval from 338.2 to 342.8 KHN were determined: placebo (no RMGIC), RMGIC without CaGP (Control), RMGIC with 1% of CaGP (1% CaGP), RMGIC with 3% of CaGP (3% CaGP) and RMGIC with 9% of CaGP (9% CaGP). The 60 block/specimen sets were subjected to a pH-cycling at 37°C for five days. The block/specimen sets were first stored for 6 hours in the demineralization solution (2.0 mmol/L Ca and P, in acetate buffer 75 mmol/L, 0.04 ppm F, pH 4.7 – 2.2 mL/mm^2^); and then for 18 hours into remineralization (1.5 mmol/L Ca, 0.9 mmol/L P, 150 mmol/L KCl, in cacodylate buffer 20 mmol/L, 0.05 ppm F, pH 7.0 – 1.1 mL/mm^2^) solution, completing one-day cycle.^15^ The block/specimen sets were always washed with distilled/deionized water for 30 seconds and dried with absorbent paper between the changes of solutions. After the 5^th^ day, the remineralization solution was renewed, in which the block/specimen sets remained for 48 hours.

### Enamel hardness analysis

After pH-cycling, the final surface hardness on enamel (SH_2_) was determined as described for SH_1_. The percentage of surface hardness loss was calculated using the following equation: %SH=((SH_2_–SH_1_)/SH_1_)×100. After SH_2_ analysis, the enamel blocks were longitudinally sectioned through the center and half of each enamel block (2×3×3 mm) was embedded in acrylic resin (Buehler Transoptic Powder, Buehler, Lake Bluff, IL, USA), and the cut surface was polished. The cross-sectional hardness was measured using 5 g load for 10 seconds from 300 µm of the contact border at depths of 5, 10, 15, 20, 25, 30, 40, 50, 70, 90, 110, 220 and 330 μm and the integrated loss of subsurface hardness (∆KHN) was calculated.^15^

### Analysis of F, Ca and P in enamel

The other halves of the enamel slabs were cut again—transversally—to obtain 2×1×3 mm slabs that were subjected to microabrasion with 400-grit silicon carbide paper in crystal polystyrene flasks.^16^ Enamel powder was dissolved in HCl 1.0 mol/L under constant shaking in an orbital shaker (TE-420 Orbital, Tecnal, Piracicaba, SP, Brazil) for 1 hour. For the analysis of total fluoride concentration, a specific electrode (Orion 9604-BN, Orion Research, Inc., Beverly, MA, USA) was used, previously calibrated with standard solutions (0.04 to 0.64 μg F/mL), and a reference microelectrode, connected to an ion analyzer (Orion 720 A, Orion Research, Inc., Beverly, MA, USA).^9^ For the Ca and P dosages, the samples were diluted (1:10) and neutralized. Ca was determined by the colorimetric method using Arsenazo III at the wavelength of 650 nm.^12^ An aliquot of 5 μL of the standards (40 to 200 μg Ca/mL) and 10 μL of the samples were plated in 96 well plates (Flat Bottom Cell Culture Plate – Model 92096 – TPP Techno Plastic Products AG, Trasadingen, Canton of Schaffhausen, Switzerland) in duplicate; then 50 μL of deionized water and Arsenazo III were added. The P was determined by the molybdate colorimetric method at the wavelength of 660 nm^17^. Aliquots of 50 μL of the samples and 100 μL of the standards (1.5 to 24 μg P/mL) in duplicate, 50 μL of molybdate and 20 μL of reducing reagent were used. The readouts were performed in duplicate using a plate reader (Microplate Spectrophotometer EON, Biotek, Winooski, VT, USA). All results were expressed in mg/mm^3^ (F, Ca and P).

## Statistical analysis

For the analysis, the material and time were considered factors of variation. The variables obtained from the release and physico-mechanical analyses showed normal distribution (Shapiro-Wilk test) and homoscedasticity (Cochran test). They were subjected to two-way analysis of variance followed by the Student-Newman-Keuls test. The values of SH_2_, %SH, ∆KHN and F, Ca and P enamel were considered pH-cycling variables, and the material, a variation factor. After confirming the homogeneous distribution, the variables were submitted to analysis of variance (1-way) followed by the Student-Newman-Keuls test. All analyses were performed using SigmaPlot version 12.0, considering statistical significance of 5%.

## Results

### F, Ca and P release from RMGIC

**Figure 3:**
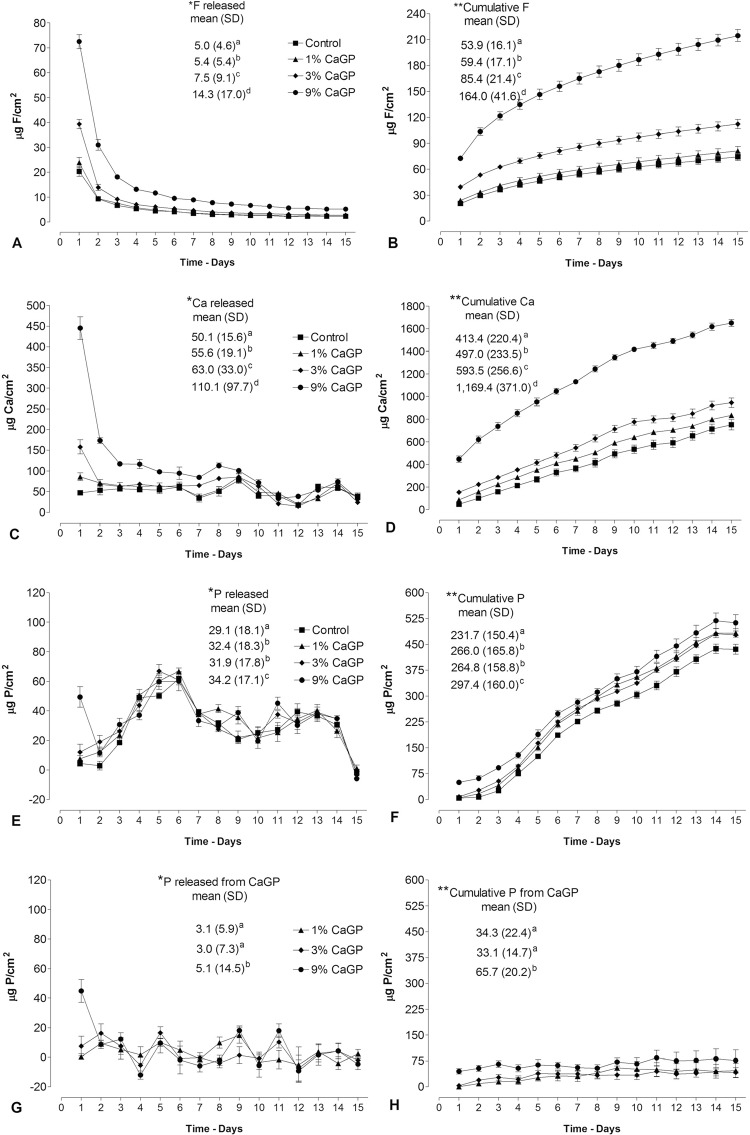
Release (mean) of F (A), Ca (C), P (E) and P of CaGP (G) in solutions (DE + RE) as function of time (15 days). Cumulative release (mean) of F (B), Ca (D), P (F) and P of CaGP (H) in solutions (DE+RE) according to the groups. Vertical bars represent standard deviation of the mean. *Mean values (SD) of total F, Ca, P and P of CaGP released. **Mean values (SD) of cumulative release of F, Ca, P and P of CaGP. Different letters indicate statistically significant difference between the groups (Student-Newman-Keuls test, p<0.001)

The highest F and Ca release values in the cycling solutions were observed on the first day for all groups (p<0.001) and decreased over time for the RMGIC containing CaGP. The group 9% CaGP presented the highest total F (Figure 3A) and Ca (Figure 3C) released values (p<0.001), followed by 3% CaGP group, respectively. From the twelfth day onwards, all groups presented constant release. The cumulative F and Ca released over 15 days (Figure 3B and 3D, respectively) was higher in the 9% CaGP (p<0.001), followed by 3% CaGP, 1% CaGP and Control group (p<0.001). The P release values were higher on the first day for 9% CaGP group (p<0.001) when compared with the other groups (Figure 3E). The highest total value was presented by 9% CaGP group (p<0.003), followed by 1 and 3% CaGP groups. All groups showed a similar release pattern, with rising and falling periods (Figure 3E). Cumulative mean of P released was higher for 9% CaGP group (p<0.001) (Figure 3F). When the values of P released by CaGP groups were subtracted from the values of the control group, the quantity of P from CaGP (Figure 3G) was noted to be higher in the group with 9% (p<0.042). Cumulative release of P from CaGP did not increase over time, but the total mean value was higher for 9% CaGP group (Figure 3H) (p<0.001). There was a positive correlation between the F and Ca released by materials (Pearson’s r=0.890; p<0.001), but there was no correlation of these ions with the P release.

### Measurements of physico-mechanical properties

The highest diametral tensile strength values were observed in the time interval of 7 days, when compared with 1 day (p<0.001), without difference between groups (p>0.060). The compressive strength did not change between the time intervals (1 day and 7 days) in all groups; notwithstanding, it was influenced by the % of CaGP. Control and 1% CaGP groups presented the highest values on the 1^st^ day (p<0.001), followed by 3% and 9% CaGP groups (Table 1). The addition of 9% of CaGP led to lower surface hardness on the 1^st^ day and higher hardness at the 7^th^ day (Table 1) when compared with the others (p<0.001). The degree of conversion did not differ between groups in the time intervals of 1 and 7 days (p>0.439).

**Table 1:** Mean values (SD) of the compressive strength, diametral tensile strength, surface hardness and degree of conversion according to the groups

Groups	Compressive strength		Diametral tensile strength		Surface hardness		Degree of conversion	
	(MPa)		(MPa)		(KHN)		(%)	
	1 day	7 days	1 day	7 days	1 day	7 days	1 day	7 days
Control	196.5^a,A*^	210.3^a,A^	16.0^a,A^	25.2^a,B^	59.7^a,A^	69.4^a,B^	64.6^a,A^	64.7^a,A^
	(13.6)	(8.7)	(5.3)	(4.7)	(2.5)	(6.9)	(3.8)	(1.6)
1% CaGP	190.6^a,A^	197.0^a,A^	13.4^a,A^	23.1^a,B^	60.6^a,A^	62.7^a,A^	65.7^a,A^	71.9^a,A^
	(15.9)	(12.5)	(2.3)	(2.7)	(4.7)	(6.5)	(2.8)	(4.5)
3% CaGP	156.9^b,A^	149.7^b,A^	9.5^b,A^	22.8^a,B^	63.5^a,A^	68.7^a,A^	61.1^a,A^	66.5^a,A^
	(7.9)	(17.9)	(1.8)	(1.0)	(5.8)	(5.4)	(6.1)	(1.6)
9% CaGP	90.3^c,A^	99.9^c,A^	7.4^b,A^	20.7^a,B^	43.9^b,A^	110.9^b,B^	62.1^a,A^	63.4^a,A^
	(11.1)	(5.7)	(0.6)	(2.6)	(3.2)	(6.9)	(0.4)	(7.5)

*Different lowercase letters indicate statistical difference between the groups and different uppercase letters indicate statistical difference between the periods of time, according to the analysis (Student-Newman-Keuls test, p<0.001)

### Demineralization and remineralization cycling

Control group presented SH_2_ and %SH values similar to that of 1% CaGP group (p>0.119); however, 1% CaGP showed lower ∆KHN values (p<0.001). The addition of 9% CaGP to RMGIC led to lower surface mineral loss values in comparison with the other groups (p<0.003), followed by 3% CaGP group (p<0.003) (Table 2). But, the mineral loss in depth did not differ between the 3% and 9% CaGP groups (p=0.106). Group 3% CaGP presented the highest F value (p<0.004); and RMGIC with 9% CaGP showed the highest value of Ca (p<0.017) present in the enamel when compared with the other groups (Table 2). Groups 3% and 9% CaGP presented similar values of P present in the enamel (p>0.399), and higher values than the other groups (p<0.022) (Table 2).

**Table 2:** Mean values (SD) of the final surface hardness (SH2), percentage surface hardness loss (%SH), integrated loss of subsurface hardness (ΔKHN), fluoride (F), calcium (Ca) and phosphate (P) from enamel according to the groups (n=12)

Groups	Analysis					
	SH_**2**_	%SH	ΔKHN	F	Ca	P
	(KHN)	(KHN)	(KHN x µm)	(µg/mm^3^)	(µg/mm^3^)	(µg/mm^3^)
Placebo	39.6^a*^	-88.3^a^	9575.0^a^	0.07^a^	621.6^a^	466.9^a^
	(14.8)	(4.5)	(510.3)	(0.01)	(111.1)	(102.1)
Control	213.7^b^	-37.1^b^	3855.4^b^	1.93^b^	606.7^a^	410.6^a^
	(29.9)	(8.7)	(714.7)	(0.63)	(149.9)	(77.9)
1% CaGP	201.4^b^	-41.1^b^	1959.5^c^	1.86^b^	743.5^a^	493.1^a^
	(23.6)	(5.8)	(636.5)	(0.39)	(188.8)	(190.8)
3% CaGP	274.7^c^	-19.5^c^	1199.3^d^	3.37^c^	974.2^b^	645.5^b^
	(16.4)	(5.9)	(275.1)	(0.79)	(157.8)	(142.6)
9% CaGP	302.3^d^	-11.1^d^	849.9^d^	2.52^d^	1276.3^c^	734.9^b^
	(17.4)	(5.1)	(320.2)	(0.93)	(413.2)	(289.3)

*Different letters indicate statistical difference between the groups according to the analysis (SH_2_, %SH, ΔKHN, F, Ca and P: Student-Newman-Keuls test, p<0.001)

## Discussion

The experimental design of this *in vitro* study allowed to verify that the addition of CaGP to RMGIC increased F release and reduced its physico-mechanical properties and mineral enamel loss. These changes were dependent on the concentration of CaGP added to the RMGIC. Thus, the null hypothesis was rejected. Although the addition of 3% CaGP had reduced the compressive strength, at the 7^th^ day it did not interfere in the other physico-mechanical properties and enhanced the resistance to demineralization by 47% when compared with the Control group.

The lower mineral loss values observed with the addition of concentrations of 3% and 9% CaGP to RMGIC were related to the higher values of F and Ca released from the materials. This phenomenon produced a medium that was supersaturated with hydroxyapatite and fluorapatite^18^ affecting mineral loss. Consequently, there was an increase in the presence of F, Ca and P in the enamel correlated to a lower mineral loss in these groups. The higher level of availability of these ions in the medium led to greater uptake by the enamel.^8^ ^,^ ^11^ The higher values of F and Ca release produced by RMGIC with CaGP could be explained by the high level of calcium availability from glycerophosphate within the matrix. Part of Ca from the CaGP probably arose from the reaction with the anionic chains of polyacids in the same way as occurs with Ca from calcium fluoroaluminosilicate particles. It could be inferred that during the acid-base reaction this would reduce the attack by the polyacids on glass particles, which would affect the release of F; or that the large amount of Ca would lead to calcium fluoride formation in the matrix, thereby retaining the F.^5^ The effect against mineral loss was also observed when the source of Ca and P was CPP-ACP.^5^ ^,^ ^19^ ^,^ ^20^ Although the explanation is related to the release of F, Ca and P, the results of F release are conflicting: or reducing,^5^ or not changing,^21^ or increasing of F release.^19^ Although the explanation given by studies was the same, complexing of F by CPP-ACP,^5^ ^,^ ^19^ the different type of GIC and methodology utilized in the studies may also have influenced the results. Moreover, the release solutions do not consider the ionic composition of the saliva and the possibility of changes in pH.^5^ ^,^ ^19^ ^,^ ^21^ Notwithstanding, an increase in the release of F and Ca was observed with the increase in concentration of CaGP in the RMGIC. Unlike GIC, the CaGP in the RMGIC promptly release the Ca that would bind to the F forming CaF^+^ ions, easily released to the environment. This statement was supported by the strong correlation between the release of F and Ca by the materials.

The P release was not correlated with Ca and F release, because it presented a different pattern of release. The pattern observed was a result of the P absorption from the DE-RE solutions, because there was no P in the composition of the ionomer, with periods of absorption and release of P (Figure 3E and 3G). The negative values indicate a reducing of P in the DE and Re solutions, since it was absorbed by materials. The calculation is based on the amount of pre-existing P in the DE and RE solutions. This same pattern could be observed for Ca from the 7^th^ day of experiment (Figure 3B), but this did not occur with F, because the DE-RE solutions did not contain this ion (Figure 3A). When subtracting the values of P released by RMGIC groups with CaGP from values of control group, little ion release was observed (Figure 3G and 3H), proving that the P released came from the DE-RE solutions. Data from cumulative release also help to confirm (Figure 3H) that the glycerophosphate was probably trapped in the matrix through the Ca binding to the polyacid, as particles in the polysalt matrix. The changes observed in the physico-mechanical properties, especially with the concentration of 9% CaGP, were due to the fact that organic phosphate interfered with the acid-base reaction, since the monomer’s degree of conversion was not altered with the addition of CaGP.

The addition of CaGP caused a decrease of the mechanical properties on 1^st^ day, particularly at the 9%concentration. According to the literature, the highest values found for diametral tensile strength (15.7 – 35.9 MPa),^22^ ^-^ ^24^ compressive strength (137.7 – 228.2 MPa)^22^ ^-^ ^25^ and hardness (64.7 – 89.4 KHN)^26^ ^,^ ^27^ were analyzed 1^st^ day after sample preparation. In this study, the values obtained (Table 1) for the Control group and RMGIC with 1% and 3% CaGP were close to the variation for this material, but with the addition of 9% CaGP they presented a reduction of 54% (diametral tensile), 54% (compressive strength) and 26% (hardness), after 1^st^ day. As in previous studies that added the CPP-ACP to the GIC,^21^ ^,^ ^28^ surface hardness tests were shown to be less sensitive to the addition of CaGP than tests of diametral tensile and compressive strength,^5^ mainly on 1^st^ day. It appears that the additions of Ca-P source over 3% produce much alteration in the composition of the powder and the powder:liquid ratio.^5^The better outcomes of diametral tensile and compressive strength and F release obtained with addition of 1.5% CPP-ACP by Mazzaoui, et al.^19^ (2003) is probably due to incorporation of low concentration, as observed by the RMGIC with 1% and 3% CaGP in the present study. Between the 1^st^ and the 7^th^ day, the mechanical properties of the RMGIC increased, because of the late acid-base reaction that occurred within the material.^29^ The addition of CaGP delayed the reaction between the polyacrylic acid and the glass particles, because glycerophosphate would consume H^+^ and increase the amount of Ca^2+^ in the acid-base reaction on the 1^st^ day. Glycerophosphate acted as a filler in the matrix together with the calcium fluorosilicate, since part of the powder was replaced by organic phosphate. Thereby, the effect on reducing mineral loss was caused by the high values of Ca and F released into the medium, and not due to adsorption of CaGP onto the enamel surface, as observed in previous studies.^9^ ^,^ ^10^

Although 9% CaGP had less ability to reduce mineral loss, the addition of 3% CaGP to the RMGIC produced better results than those of the Control group. This was confirmed by the greater presence of F in enamel, and by the 60% increase in Ca and P in comparison with the Control group. In 9% CaGP group, the presence of higher Ca values together with the increase in F and P produced lower mineral loss because of higher release of Ca and F from the material. However, a higher level of availability of Ca in the medium may lead to less presence of F in the enamel^10^, as observed in this study, when 3% and 9% CaGP groups were compared (Table 2). Thus, an appropriate amount of organic phosphate can be added to the resin-modified glass ionomer powder, thereby improving its effect against demineralization with minimal changes in its physico-mechanical properties.

## Conclusion

It was concluded that the incorporation of 3% CaGP into RMGIC increased the release of Ca and F and reduced enamel demineralization, thereby maintaining the physico-mechanical properties within the parameters for this material.
